# Tetanus-diphtheria vaccine can prime SARS-CoV-2 cross-reactive T cells

**DOI:** 10.3389/fimmu.2024.1425374

**Published:** 2024-07-18

**Authors:** Sara Alonso Fernandez, Hector F. Pelaez-Prestel, Tara Fiyouzi, Marta Gomez-Perosanz, Jesús Reiné, Pedro A. Reche

**Affiliations:** ^1^ Department of Immunology & O2, Faculty of Medicine, Complutense University of Madrid, Ciudad Universitaria, Madrid, Spain; ^2^ Clinical Sciences, Liverpool School of Tropical Medicine, Liverpool, United Kingdom; ^3^ Oxford Vaccine Group, University of Oxford, Oxford, United Kingdom

**Keywords:** COVID-19, SARS-CoV-2, epitope, T cell cross-reactivity, tetanus-diphtheria toxoid vaccines

## Abstract

Vaccines containing tetanus-diphtheria antigens have been postulated to induce cross-reactive immunity to severe acute respiratory syndrome coronavirus 2 (SARS-CoV-2), which could protect against coronavirus disease (COVID-19). In this work, we investigated the capacity of Tetanus-diphtheria (Td) vaccine to prime existing T cell immunity to SARS-CoV-2. To that end, we first collected known SARS-CoV-2 specific CD8^+^ T cell epitopes targeted during the course of SARS-CoV-2 infection in humans and identified as potentially cross-reactive with Td vaccine those sharing similarity with tetanus-diphtheria vaccine antigens, as judged by Levenshtein edit distances (≤ 20% edits per epitope sequence). As a result, we selected 25 potentially cross-reactive SARS-CoV-2 specific CD8^+^ T cell epitopes with high population coverage that were assembled into a synthetic peptide pool (TDX pool). Using peripheral blood mononuclear cells, we first determined by intracellular IFNγ staining assays existing CD8^+^ T cell recall responses to the TDX pool and to other peptide pools, including overlapping peptide pools covering SARS-CoV-2 Spike protein and Nucleocapsid phosphoprotein (NP). In the studied subjects, CD8^+^ T cell recall responses to Spike and TDX peptide pools were dominant and comparable, while recall responses to NP peptide pool were less frequent and weaker. Subsequently, we studied responses to the same peptides using antigen-inexperienced naive T cells primed/stimulated *in vitro* with Td vaccine. Priming stimulations were carried out by co-culturing naive T cells with autologous irradiated peripheral mononuclear cells in the presence of Td vaccine, IL-2, IL-7 and IL-15. Interestingly, naive CD8^+^ T cells stimulated/primed with Td vaccine responded strongly and specifically to the TDX pool, not to other SARS-CoV-2 peptide pools. Finally, we show that Td-immunization of C57BL/6J mice elicited T cells cross-reactive with the TDX pool. Collectively, our findings support that tetanus-diphtheria vaccines can prime SARS-CoV-2 cross-reactive T cells and likely contribute to shape the T cell responses to the virus.

## 1 Introduction

Severe acute respiratory syndrome coronavirus 2 (SARS-CoV-2) is an emergent β-coronavirus identified in late 2019, causing pneumonia as well as a wide array of ailments and symptoms under the umbrella of coronavirus disease 2019 (COVID-19) (1). The rapid spread and pathogenesis of SARS-CoV-2 resulted in a global pandemic and health crisis that urged the mass deployment of novel COVID-19 vaccines (2). However, SARS-CoV-2 remains a public health concern since COVID-19 vaccines do not provide sterile immunity (3) and new SARS-CoV-2 variants have emerged (4). Hence, effective and universal measures against COVID-19 are still in demand. Fortunately, we now know much about SARS-CoV-2 infection and immune responses, which should help in this endeavor.

It is now clear that exposure to SARS-CoV-2 does not always result in infection, nor does infection follow the same course in everyone (5). Several factors have been identified to increase the severity of COVID-19, most notably old age, but also obesity, male gender and the presence of conditions like diabetes and vascular diseases (6). The immune response to SARS-CoV-2 plays itself a major role in the course of infection. The most severe cases of COVID-19 are characterized by significant immunopathology, resulting from a disproportionate anti-viral innate immune response, concomitant with a poor adaptive immune response (7, 8). In contrast, COVID-19 severity and duration are reduced in individuals developing a coordinated adaptive immune response, involving SARS-CoV-2-specific CD4^+^ and CD8^+^ T cells and neutralizing antibodies (9). However, anti-SARS-CoV-2 neutralizing antibodies are short-lived (10) and long-term immunity to SARS-CoV-2 appears to be mediated by memory T cells (11–13). Moreover, there is evidence indicating that T cell immunity alone, in the absence of neutralizing antibodies, may protect from SARS-CoV-2 (14). SARS-CoV-2-specific T cell immunity is characterized by CD4^+^ T cells with a typical T cell helper type 1 (Th1) phenotype (11, 13) but displaying a lower IFNγ/TNFα ratio than influenza-specific Th1 responses (15). Th1 cells promote the activation and differentiation of SARS-CoV-2 specific cytotoxic CD8^+^ T cells, which are crucial for resolving the infection by killing infected cells (16, 17). In fact, delayed CD8^+^ T cell responses have been linked to severe COVID-19, since viral replication in the lungs is not controlled sufficiently fast (18).

Adaptive immune responses to SARS-CoV-2 induced by both infection and vaccines are surely influenced by pre-existing cross-reactive immunity (19–22). Cross-reactive immunity occurs when memory T and B cells elicited by a primary encounter with pathogens/antigens recognize and respond to different pathogens/antigens (23, 24). The first evidence of pre-existing cross-reactive immunity to SARS-CoV-2 came from the extensive T cell and antibody responses to the virus detected in unexposed individuals prior to or early in the outbreak (25–28). T cells are by nature more cross-reactive than B cells (29) and about 20–85% of unexposed individuals have been shown to present T cell reactivity to SARS-CoV-2 (25, 30). Interestingly, while pre-existing B-cell cross-reactivity can enhance SARS-CoV-2 pathogenesis (31, 32), cross-reactive memory T cells contribute to host protection (33).

Immune cross-reactivity is more likely to occur, and easy to detect, between related pathogens/antigens. SARS-CoV-2 shares sequence and structure similarity with common cold human coronavirus (ccHCoVs), comprising two α-coronaviruses (299E and NL63) and two β-coronaviruses (HKU1, OC43) (34), which cause seasonal and prevalent infections in humans (35, 36). Therefore, immune cross-reactivity between SARS-CoV-2 and ccHCoVs has received major attention and is widely documented (37, 38). It has been reported that up to 50% of T cell clones generated from unexposed subjects against SARS-CoV-2 peptides can cross-react with ccHCoVs peptides (39). However, other studies point out that the cross-reactive T cell epitope repertoire between SARS-CoV-2 and ccHCoVs is much smaller (40, 41). Moreover, T cell cross-reactivity has also been identified between SARS-CoV-2 and unrelated pathogens/antigens, including bacteria (42) and common viruses like human cytomegalovirus (43, 44) and influenza virus (44). Therefore, the priming sources of cross-reactive T cells to SARS-CoV-2 and contribution to protection are still unclear.

Adaptive immunity develops early during childhood from exposure to environmental antigenic challenges (e.g. microbes and vaccines) (45), and so does cross-reactive immunity to SARS-CoV-2. Interestingly, small children, who are generally vulnerable to new pathogens, are particularly resilient to SARS-CoV-2 infection (46). Since children receive multiple vaccinations from infancy to puberty, we investigated in a seminal *in silico* work common vaccines as potential sources of cross-reactive immunity to SARS-CoV-2 (47, 48). We concluded that vaccines containing tetanus-diphtheria antigens could induce cross-reactive protective immunity to SARS-CoV-2 (47, 48). Evidence of such protection was confirmed latter (49) and it has been shown that T cells expanded with SARS-CoV-2 antigens and Tdap vaccine, which includes tetanus-diphtheria antigens and acellular *Bordetella pertussis* antigens, exhibit overlapping T cell receptor (TCR) repertoires (50). It is worth noting that vaccines containing tetanus-diphtheria toxoids include far more proteins than the inactivated toxins. As revealed by proteomics analysis, diphtheria and tetanus toxoids only account for ~50–70% of the total protein content in these vaccines, being accompanied by hundreds of additional proteins from the relevant bacteria (51–53). All these tetanus-diphtheria antigens can be immunogenic and were taken into consideration in our former *in silico* analysis. Given the relevance of CD8^+^ T cells in clearing viral infections, in this work, we experimentally studied SARS-CoV-2 CD8^+^ T cell cross-reactivity from tetanus-diphtheria Td vaccines. We report that stimulation of naive T cells with autologous irradiated peripheral mononuclear cells pulsed with a tetanus-diphtheria Td vaccine renders them cross-reactive with a peptide pool consisting of 25 known SARS-CoV-2-specific CD8^+^ T cell epitopes related by similarity with antigens in Td vaccines (TDX pool). In contrast, these same T cells seldom responded to control peptides, including other SARS-CoV-2 peptide pools from Spike protein and Nucleocapsid phosphoprotein (NP). In addition, we also found that Td immunization of C57BL/6J mice induced T cell responses to the TDX pool. These results support that tetanus-diphtheria vaccines can prime SARS-CoV-2 cross-reactive T cells and likely contribute to shape the T cell responses to the virus.

## 2 Methods

### 2.1 Selection of cross-reactive SARS-CoV-2-specific CD8^+^ T cell epitopes, prediction of binding to MHC I molecules and computation of population coverage

SARS-CoV-2-specific CD8^+^ T cell epitopes potentially cross-reactive with tetanus-diphtheria antigens were selected upon experimentally verified SARS-CoV-2-specific CD8^+^ T cell epitopes targeted by humans infected with SARS-CoV-2. Such SARS-CoV-2-specific CD8^+^ T cell epitopes were obtained from the Immune Epitope Database (IEDB)[ (54) after the following search criteria: 1) Peptide, linear; 2) Host, human; 3) Source, SARS-CoV-2; 4) T cell assay, positive results only; 5) Restriction, Class I and 6) Disease, infection. T cell epitope assays were downloaded and processed, and a dataset consisting of the amino acid sequences of 1153 distinct SARS-CoV-2-specific CD8^+^ T cell epitopes with their reported restriction elements was assembled (**Supplementary Dataset 1**). Subsequently, a PERL script for fuzzy matching based on Levenshtein edit distances (String: Approx perl extension) was used to select CD8^+^ T cell epitopes whose sequences matched those of antigens identified in tetanus-diphtheria vaccines. Approximate matches of up to 20% edits per epitope sequence (insertions, deletions or substitutions) were allowed. A 20% Levenshtein distance for a peptide of 10 residues means that two editions are required to produce a match, while a peptide matching exactly has 0 Levenshtein distance. The Levenshtein distance between sequences is related to their similarity but it does not align with a fixed percentage of similarity. Protein antigens in diphtheria and tetanus toxoid vaccines were those identified through proteomics studies and available in proteome datasets PXD012806 (51), PXD013804 (52) and PXD009289 (53) at the Proteomics Identification Database (PRIDE). Protein sequences were retrieved from UniProt and assembled into a single file in FASTA format including 210 antigens from *Corynebacterium diphtheriae* (diphtheria) and 548 from *Clostridium tetani* (tetanus). Antigen sequences and PERL script for fuzzy matching can be obtained from the corresponding author upon written request.

Binding of CD8^+^ T cell peptide epitopes to human and mouse major histocompatibility complex class I (MHC I) molecules was predicted using standalone versions of RANKPEP (55, 56) and NetMHCpan (57, 58). The targeted MHC I molecules included 22 human leukocyte antigens class I (HLA I) molecules (HLA-A*01:01, HLA-A*02:01, HLA-A*02:03, HLA-A*02:06, HLA-A*03:01, HLA-A*11:01, HLA-A*23:01, HLA-A*24:02. HLA-A*26:01, HLA-A*30:01, HLA-A*30:02, HLA-A*31:01, HLA-A*32:01, HLA-A*33:01, HLA-A*68:01, HLA-A*68:02, HLA-B*07:02, HLA-B*08:01, HLA-B*15:01, HLA-B*35:01, HLA-B*40:01 and HLA-B*44:02) and 9 mouse class I H2 alloantigens (H2-Db, H2-Dd, H2-Dq, H2-Kb, H2-Kd, H2-Kk, H2-Kq, H-2-Ld and H-2-Lq). RANKPEP and NetMHCpan prediction models were selected to match the size of the peptides that had 8 or 9 residues. For longer peptides, the % rank of all nested 9mer peptides was analyzed and the best rank assigned to the peptide. Peptides were considered to bind to any given MHC I molecule if they were reported to have a % rank ≤ 2 by either RANKPEP or NetMHCpan. Human population coverage of CD8^+^ T cell epitopes was computed after HLA I binding profiles using a standalone version of EPISOPT (59).

### 2.2 Synthetic peptides and peptide pools

Synthetic peptides corresponding to SARS-CoV-2-specific CD8^+^ T cell epitopes cross-reactive with tetanus-diphtheria antigens were obtained from ProteoGenix at 2 mg scale and ≥ 90% purity. Peptides were dissolved in 80% dimethyl sulfoxide (DMSO), diluted to a final stock concentration of 5 mM (40% DMSO) and stored at -80°C. A custom peptide pool (TDX pool) was prepared by combining an equal volume of all these peptides (final concentration 200 µM). Commercial SARS-CoV-2 peptide pools consisting of overlapping peptides spanning the entire SARS-CoV-2 nucleocapsid phosphoprotein (NP pool) and S1 immunogenic region of Spike protein (Spike pool) were purchased from Miltenyi: PepTivator^®^ SARS-CoV-2 Prot_N and PepTivator^®^ SARS-CoV-2 Prot_S, respectively (reference Wuhan strain). CEF peptide pool consisting of immunodominant CD8^+^ T cell peptide epitopes from Human Cytomegalovirus, Epstein-Barr and Influenza A viruses was purchased from Mabtech. PepTivators^®^ pools were reconstituted in sterile H_2_O (30 µM final concentration) and CEF pool in DMSO plus phosphate-buffered saline (PBS) buffer (200 µg/ml final concentration), following the manufacturer’s instructions.

### 2.3 Culture media and reagents

Human cells were cultured in RPMI complete medium consisting of RPMI 1640 medium (Gibco) supplemented with 10% of heat-inactivated human serum (Gibco), 2 mM L-glutamine (Lonza), and 100 U/ml penicillin (Lonza) and 100 μg/ml streptomycin (Lonza). Splenocytes from mice were also incubated in RPMI complete medium, but including 10% of heat inactivated fetal bovine serum (FBS)(Gibco), instead of human serum. Cytokines for cell cultures were obtained from Immunotools GmbH. DIFTAVAX^®^ Tetanus-diphtheria (Td) toxoids vaccine (Sanofi-Pasteur) was used for *in vitro* stimulations and *in vivo* immunizations. DIFTAVAX^®^ (Td) contains no less than 2.5 Lf (2 IU) of purified diphtheria toxoid and 5 Lf (20 IU) of purified tetanus toxoid per dose (0.5 ml).

### 2.4 Isolation of peripheral blood mononuclear cells and naive T cells

Peripheral blood mononuclear cells (PBMCs) were isolated from buffy coats by a density gradient on Ficoll-Paque™ PLUS (Fisher Scientific). PBMCs in the interface layer were collected, washed twice with cold PBS, resuspended in complete RPMI medium and counted. Buffy coats were provided by the regional blood transfusion center (Centro de Transfusión de la Comunidad de Madrid, Spain), and were obtained from healthy donors after written informed consent. Naive T cells were isolated from PBMCs by negative selection using a magnetic separation kit (EasySepTM Human Naive Pan T Cell Isolation, StemcellTM Technologies). Briefly, freshly isolated PBMCs (~5x10^7^ cells) were incubated in PBS containing 2% of heat-inactivated human serum and 1 mM EDTA (1ml) with T cell isolation and TCR Gamma/Delta depletion antibody cocktails (50 µl each) for 5 minutes, and then with magnetic beads (60 µl) capturing antibody-labeled cells for 3 minutes. Magnetic-labeled cells were then pulled out with the help of a magnet, leaving untouched isolated naive T cells in the media. All isolation steps were performed at room temperature. To control the purification, freshly isolated naive T cells (~10^5^ cells) were stained with anti-human CD3-APC (UCHT1, BD Biosciences), anti-human CD45RA-PE (HI100, BD Biosciences) and anti-human CD45RO-FITC (UCHL1, Miltenyi Biotec) antibodies, and analyzed by flow cytometry. On average, 5x10^6^ cells were isolated from 5x10^7^ PBMCs.

### 2.5 T cell proliferation assay

Proliferation of T cells was determined by Carboxyfluorescein Diacetate Succinimidyl Ester (CFSE)(Biolegend) dilution assay, which was used as a criterium to select an optimal working concentration of Td vaccine. About 10^7^ PBMCs were incubated with CSFE (0.5 µM final concentration) for 20 min in PBS at 37°C. Cells were washed twice using complete RPMI and plated on 96-well cell-culture plates (10^5^ cells/well) with IL-2 (20 ng/ml) and varying concentrations of Td vaccine as determined by the content of diphtheria toxoid (0.0012, 0.004 and 0.04 Lf/ml of diphtheria toxoid). Plates were incubated at 37°C and 5% CO_2_ for 5 days. As controls, PBMCs were incubated with 25 ng/ml phorbol 12-myristate 13-acetate (PMA)(Merck) or media alone. CFSE-labeled cells were then stained with anti-human CD3-APC antibody (UCHT-1, BD Biosciences) and analyzed by flow cytometry.

### 2.6 Stimulation of naive T cells with Td vaccine

Naive T cells were primed with diphtheria-tetanus antigens using irradiated PBMCs pulsed with Td vaccine. About 10^7^ PBMCs at a density of 5x10^6^ cells/ml were incubated with Td vaccine (0.0012 Lf/ml) for 30 min in a sterile 15 ml tube with 2 ml of complete RMPI media. PBMCs were then homogenously irradiated with 30 Gy (Gammacell 1000 irradiator, Nordion). Td-pulsed irradiated PBMCs were disposed in p24 plates (4x10^5^ cells/well) along with 4x10^5^ of autologous naive T cells per well in complete RPMI (800 μl) supplemented with Td vaccine (0.0012 lf/ml) plus IL-2 (20 ng/ml), IL-7 (25 ng/ml) and IL-15 (25 ng/ml) (cytokines from ImmunoTools) and were incubated for 13 days at 37°C and 5% CO_2_. Td vaccine and cytokines were renewed every 2 days and 200 µl of growth medium replenished.

### 2.7 Mice immunizations and preparation of splenocytes

All mice procedures included in this study were reviewed and approved by the Ethics Board Committee at the Universidad Complutense de Madrid and by the Division of Animal Protection of the Comunidad de Madrid. C57BL/6J mice (male, 6 weeks old, Charles River) received 3 intramuscular (IM) immunizations at 3-week intervals with 1/25 dose of Td vaccine diluted in 100 μl of PBS (Td vaccinated group, n = 5) or with PBS alone (control group, n = 5). Seven days after the last immunization, mice were sacrificed by cervical dislocation, under general anesthesia with 1–2% isoflurane/O_2_. At termination, blood was obtained from mice via cardiac puncture, collecting 0.2 ml in 1.5 ml microcentrifuge tubes (Eppendorf). The samples were then centrifuged at 10,000 rpm for 10 minutes at room temperature. Afterward, serum was carefully collected and stored at -80°C for subsequent analysis. To examine mice immunization with Td vaccine, the concentration of specific tetanus toxoid (TT) IgG was quantified by an indirect ELISA assay using Tetanus Toxoid Coated Plates (Biomat). The plates were incubated with serum samples (1:400) for 1 hour at room temperature, and after 3 washes with PBS containing Tween 20 (Sigma-Aldrich), incubated with HRP-conjugated IgG1 secondary anti-mouse antibody (PA1–74421, Invitrogen) (1:4000) followed by the addition of TMB substrate. The reaction was stopped with 1 M HCl stop solution and optical density (OD) was measured at 492 nm using a BioTek plate reader (Agilent). The average blank corrected value was calculated for each sample, and the data was analyzed using BioTek Gen5 software (Agilent). Spleens were also collected and processed as follows. Spleens were minced and filtered through 70 µm nylon cell strainers (Corning) to obtain a single-cell suspension. Cells were washed with cold PBS containing 2% FBS and red blood cells lysed in ammonium-chloride-potassium (ACK) lysis buffer (Gibco). The remaining splenocytes were washed 2 times with cold PBS containing 2% FBS, counted and resuspended in complete RPMI at a density of 2x10^6^ cells/ml.

### 2.8 Detection of peptide-specific CD8^+^ T cell responses

CD8^+^ T cell responses to peptides pools (SARS-CoV-2 TDX, NP and Spike pools, and control CEF pool) were determined by intracellular IFNγ staining using human PBMCs (recall response), Naive T cells stimulated with Td vaccine and splenocytes from mice (Td immunized and controls). Human cells (PBMCs and T cells) in complete fresh RPMI were plated in 24-well plates (1x10^6^ cells/well), rested for 30 minutes and then cultured at 37°C and 5% CO2 for 16 hours with the relevant peptide pools and a Golgi inhibitor (Brefeldin A) at 2.5 μg/mL (Thermo Fisher Scientific). A negative control condition consisting of media alone with DMSO (0.3%) was also included. Mouse splenocytes in complete RPMI (2x10^6^ cells/well) were cultured in 24-well plates with the relevant peptides or media with DMSO (0.3%) for 36 hours at 37°C and 5% CO_2_. Brefeldin A (2.5 μg/ml) was added the last 16 hours of culture. SARS-CoV-2 TDX pool in cell cultures was at 2.0 µM (each peptide) and SARS-CoV-2 NP and Spike pools were at 0.6 µM (each peptide, as recommended by the manufacturer). CEF pool was used at a final concentration of 2 µg/ml, following the manufacturer’s recommendations. After peptide stimulations, cells were stained with anti-human CD3-PE (UCHT1, Biolegend) or anti-mouse CD3-PE (17A2, Biolegend) and anti-human CD8-FITC (SK1, Biolegend) or anti-mouse CD8-FITC (Ssa1, ImmunoTools). Subsequently, cells were permeabilized and stained intracellularly with anti-human IFNγ-APC (B27, Biolegend) or anti-mouse IFNγ-APC (XMG1.2, BD Biosciences). Finally, cells were acquired and analyzed by flow cytometry, and CD3^+^CD8^+^IFNγ^+^ cells quantified. In these intracellular IFNγ staining assays, the positive IFNγ^+^ gate was set utilizing Fluorescence Minus One (FMO) controls. These controls and the delimitation of the gate were obtained after stimulating human PBMCs and mouse splenocytes with Phytohemagglutinin-L as a positive control (PHA-L, Sigma)(**Supplementary Figure S1**).

### 2.9 Flow cytometry general procedures

Cells were washed twice with PBS prior to any staining and with ZombieAqua for live/dead cell discrimination (Biolegend). For surface staining, Fc receptors were first blocked with 200 µg/ml of human IgG from human serum (Merck). Next, cells were stained with the relevant antibodies diluted 1:25 in PBS supplemented with 0.5% of FBS and 1 mM EDTA (50 μL of final volume/sample), incubating for 30 minutes at room temperature. Finally, cells were fixed with BD CytofixTM (BD Biosciences), containing 4.2% formaldehyde, unless intracellular staining for IFNγ detection was performed (described earlier). After staining, cell samples were washed twice in PBS and resuspended in PBS with 1 mM EDTA (200 μl of final volume/sample). Cells were acquired on BD FACSCelesta and FACSCalibur flow cytometers (BD Biosciences) (human samples and mouse samples, respectively), and analyzed using FlowJo software (version 10, Treestar). Compensation matrices were set using compensation beads (BD Biosciences) and ArC™ Amine Reactive Compensation beads (Thermofisher). For data analysis, we performed live/dead cell discrimination on single cells, and subsequently gated on the relevant staining.

### 2.10 Statistical analyses

Kruskal-Wallis tests were used for comparing T cell responses to different peptide pools and media in human and mice samples. Wilcoxon signed-rank tests were applied to compare recall and Td-primed T cell responses to the same peptide pools in the same subjects. Mann-Whitney U tests were used to compare T cell responses to the same peptides between groups of immunized and control mice. *p* < 0.05 was considered significant. Statistic calculations were performed on GraphPad Prism 8.

## 3 Results

### 3.1 SARS-CoV-2-specific CD8^+^ T cell epitopes with similarity to tetanus-diphtheria vaccine antigens

We identified CD8^+^ T cells epitopes potentially cross-reactive with tetanus-diphtheria vaccine antigens within a set of known SARS-CoV-2-specific CD8^+^ T cell epitopes (**Supplementary Dataset 1**). This set consisted of 1153 experimentally verified CD8^+^ T cell epitopes, recognized by humans infected with SARS-CoV-2 (details in Methods). To identify potentially cross-reactive CD8^+^ T cell epitopes, we relied on Levenshtein edit distances to detect sequence similarity to tetanus-diphtheria vaccine antigens. In particular, SARS-CoV-2-specific CD8^+^ T cell epitopes matching tetanus-diphtheria vaccine antigens with ≤ 20% edit distances were considered as potentially cross-reactive. We found that 66 SARS-CoV-2-specific CD8^+^ T cell epitopes met this criterion (**Supplementary Dataset 2**) and selected 25 for experimental analyses (**Table 1**). The selection of CD8^+^ T cell epitopes was made to cover the maximum number of SARS-CoV-2 antigens and HLA I molecules. The selected CD8^+^ T cell epitopes span over 10 distinct SARS-CoV-2 mature antigens with the majority lying on the Spike (8 epitopes) and Polymerase (POL)(5 epitopes) proteins. These epitopes are collectively noted to be restricted by 13 distinct HLA I molecules. Judging by the phenotypic frequency of these HLA I molecules, over 85% of the population, regardless of ethnicity, could respond to any of these CD8^+^ T cell epitopes (See Methods). This population coverage is likely to be much greater and to reach the entire population because many more HLA I molecules are predicted to present these CD8^+^ T cell epitopes (**Table 1**). We also predicted that some of these CD8^+^ T cell epitopes could be presented by mouse class I H2 alloantigens (**Table 1**). To experimentally address cross-reactivity, the selected SARS-CoV-2-specific T cell epitopes were synthesized and combined in a peptide pool (TDX pool).

**Table 1:** Potentially cross-reactive SARS-CoV-2-specific CD8^+^ T cell epitopes with tetanus-diphtheria vaccine antigens.

Epitope Sequence	Antigen [NCBI Accession]	HLA I Presentation (experimental)	HLA I presentation (Predicted)	H2 I presentation (Predicted)	Td Peptide^1^	Td antigen^2^ ACC|[T/D]	Td peptide HLA I presentation^3^ (Predicted)
IIWVATEGA	NP [YP_009724397]	HLA-A*02:01	NN	NN	IIKVATEDG	Q897I8|T	NN
QLNRALTGI	SPIKE [YP_009724390]	HLA-A*02:03	HLA-A*02:01, HLA-A*02:03	NN	QLREALTGI	Q895W2|T	HLA-A*02:01, HLA-A*02:03, HLA-A*02:06, HLA-A*30:01
FERDISTEI	SPIKE [YP_009724390]	HLA-B*40:01	HLA-B*40:01, HLA-B*44:02, HLA-B*44:03, HLA-B*51:01	H-2-Kk, H-2-Kq, H-2-Lq	FMRDIDAEI	Q894X4|T	HLA-A*02:01, HLA A*02:03, HLA-A*02:06, HLA-B*08:01, HLA-B*15:01, HLA-B*44:03
SFELLHAPATV	SPIKE [YP_009724390]	HLA-A*02:01	HLA-A*02:01, HLA-A*02:03, HLA-A*02:06, HLA-A*68:02, HLA-B*40:01	H-2-Kd, H-2-Kk	AFELLHACPQV	Q6NJ45|D	HLA-A*02:01, HLA-A*02:03, HLA-A*02:06, HLA-A*68:02
ATVVIGTSK	POL [YP_009725307]	HLA-A*11:01	HLA-A*03:01, HLA-A*11:01, HLA-A*30:01, HLA-A*31:01, HLA-A*68:01	NN	ATVAEGTKK	Q6NF63|D	HLA-A*03:01, HLA-A*11:01, HLA-A*30:01, HLA-A*68:01
AQALNTLVKQL	SPIKE [YP_009724390]	HLA class I	HLA-A*02:01, HLA-A*02:03, HLA-A*02:06, HLA-A*03:01, HLA-A*11:01, HLA-A*30:01, HLA-A*32:01, HLA-B*08:01	H-2-Db, H-2-Kd	VAALNGLVKQG	Q6NG46|D	HLA-A*03:01, HLA-A*11:01, HLA-B*51:01
IVAGGIVAI	NSP4 [YP_00972530]	HLA-A*02:01	HLA-A*02:01, HLA-A*02:03, HLA-A*02:06, HLA-A*26:01, HLA-A*32:01, HLA-A*68:02, HLA-B*51:01	NN	IVAGGGVAL	Q891Q6|T	HLA-A*02:01, HLA-A*02:03, HLA-A*02:06, HLA-A*26:01, HLA-A*32:01, HLA-A*68:02, HLA-B*07:02, HLA-B*15:01, HLA-B*35:01
FVFKNIDGY	SPIKE [YP_009724390]	HLA-A*29:02, HLA-A*26:01	HLA-A*01:01, HLA-A*26:01, HLA-A*30:02, HLA-A*32:01, HLA-A*68:01, HLA-B*15:01, HLA-B*35:01, HLA-B*53:01	NN	QKFVNIDGY	Q891E4|T	HLA-A*30:02
IMASLVLAR	POL [YP_009725307]	HLA-A*33:01	HLA-A*03:01, HLA-A*11:01, HLA-A*31:01, HLA-A*33:01, HLA-A*68:01	NN	IFASLYLAR	Q895E4|T	HLA-A*31:01, HLA-A*33:01
ILRGHLRIA	MP [YP_009724393]	HLA-A*02:03	HLA-A*02:03, HLA-A*30:01	NN	KLALHLRIA	Q6NH14|D	HLA-A*02:03, HLA-A*30:01
MASLVLARK	POL [YP_009725307]	HLA-A*68:01	HLA-A*03:01, HLA-A*11:01, HLA-A*30:01, HLA-A*33:01, HLA-A*68:01	NN	VASLVSALK	Q899H3|T	HLA-A*03:01, HLA-A*11:01, HLA-A*30:01, HLA-A*68:01, HLA-B*51:01
LVKPSFYVY	ENV [YP_00972439]	HLA-C*07:02	HLA-A*01:01, HLA-A*03:01, HLA-A*11:01, HLA-A*26:01, HLA-A*30:01, HLA-A*30:02, HLA-A*32:01, HLA-B*15:01, HLA-B*35:01, HLA-B*53:01, HLA-B*57:01, HLA-B*58:01	NN	EVKPSSYVY	Q893Q3|T	HLA-A*01:01, HLA-A*26:01, HLA-A*30:02, HLA-A*32:01, HLA-A*33:01, HLA-A*68:01, HLA-A*68:02, HLA-B*15:01, HLA-B*35:01, HLA-B*44:02, HLA-B*44:03, HLA-B*53:01
FVAAIFYLI	NSP4 [YP_00972530]	HLA-A*02:01	HLA-A*02:01, HLA-A*02:03, HLA-A*02:06, HLA-A*68:02, HLA-B*51:01	H-2-Db, H-2-Dd, H-2-Kb	IFAAIMYLI	Q895R4|T	HLA-A*23:01, HLA-A*24:02, HLA-B*51:01
TLADAGFIK	SPIKE [YP_009724390]	HLA-A*03:01	HLA-A*03:01, HLA-A*11:01, HLA-A*68:01	NN	TLDAGFIPR	Q6NG84|D	HLA-A*03:01, HLA-A*11:01, HLA-A*31:01, HLA-A*33:01, HLA-A*68:01
LLDRLNQL	NP [YP_009724397]	HLA class I	HLA-A*02:01, HLA-A*02:03, HLA-B*08:01	NN	NLDKLNQL	Q897F3|T	HLA-B*08:01
AYSNNSIAI	SPIKE [YP_009724390]	HLA-A*24:02	HLA-A*23:01, HLA-A*24:02	H-2-Db, H-2-Kd	AYSHYSIAI	Q6NJH2|D	HLA-A*23:01, HLA-A*24:02, HLA-B*51:01
IPTITQMNL	POL [YP_009725307]	HLA-B*07:02	HLA-B*07:02, HLA-B*08:01, HLA-B*35:01, HLA-B*51:01, HLA-B*53:01	H-2-Dq, H-2-Ld, H-2-Lq	IPTIFQDNL	Q6NFM0|D	HLA-B*07:02, HLA-B*35:01, HLA-B*51:01, HLA-B*53:01
TDLEGNFY	3CPR [YP_009725301]	HLA-A*01:01	HLA-A*01:01, HLA-A*26:01, HLA-A*30:02	NN	LDDEGNFY	Q893J1|T	HLA-A*01:01
VTNNTFTLK	NSP2 [YP_009725298]	HLA-A*03:01, HLA-A*11:01	HLA-A*03:01, HLA-A*11:01, HLA-A*30:01, HLA-A*31:01, HLA-A*68:01	H-2-Db, H-2-Dd, H-2-Kb	DATNTFTLK	Q6NF84|D	HLA-A*03:01, HLA-A*11:01, HLA-A*33:01, HLA-A*68:01, HLA-B*51:01
TYVPAQEKNFT	SPIKE [YP_009724390]	HLA-A*24:02	HLA-A*23:01, HLA-A*24:02, HLA-A*26:01, HLA-B*35:01, HLA-B*53:01	H-2-Dd, H-2-Dq, H-2-Kd, H-2-Ld H-2-Lq	TNVHAQEKNFN	Q899V7|T	HLA-A*26:01
TDNYITTY	NSP3 [YP_009725299]	HLA-A*01:01	HLA-A*01:01, HLA-B*44:02	H-2-Kq	TINYITEY	Q898F9|T	HLA-A*01:01, HLA-A*26:01, HLA-A*30:02, HLA-B*15:01, HLA-B*35:01
KRVDWTIEY	35EXON [YP_009725309]	HLA-B*07:02	HLA-A*01:01, HLA-A*26:01, HLA-A*30:02, HLA-A*32:01, HLA-B*44:03	NN	KRVDWDIEY	Q899B2|T	HLA-A*01:01, HLA-A*30:02
TLIGDCATV	2ORMT [YP_009725311]	HLA class I	HLA-A*02:01, HLA-A*02:03, HLA-A*02:06, HLA-A*68:02	NN	TLIIDATCV	Q890S3|T	HLA-A*02:01, HLA-A*02:03, HLA-A*02:06
VLAWLYAAV	3CPR [YP_009725301]	HLA class I	HLA-A*02:01, HLA-A*02:03, HLA-A*02:06, HLA-B*51:01	H-2-Kb	VLAALGAAA	Q6NFZ1|T	HLA-A*02:03
LRIMASLVL	POL [YP_009725307]	HLA-C*07:02	NN	NN	LRAMASEVL	P62411|D	NA

NN, None predicted. Underlined epitopes lie within antigen regions covered by the Spike peptide pool. ^1^Td peptide equivalent to SARS-CoV-2 CD8^+^ T cell epitope. ^2^UniProt accession number (ACC) of Td peptide protein source followed by T or D, indicating a Tetanus or Diphtheria antigen,respectively. ^3^Predicted HLA I presentation profile of Td peptide.

### 3.2 Detection of existing T cell responses to SARS-CoV-2 TDX pool

**Figure 1:**
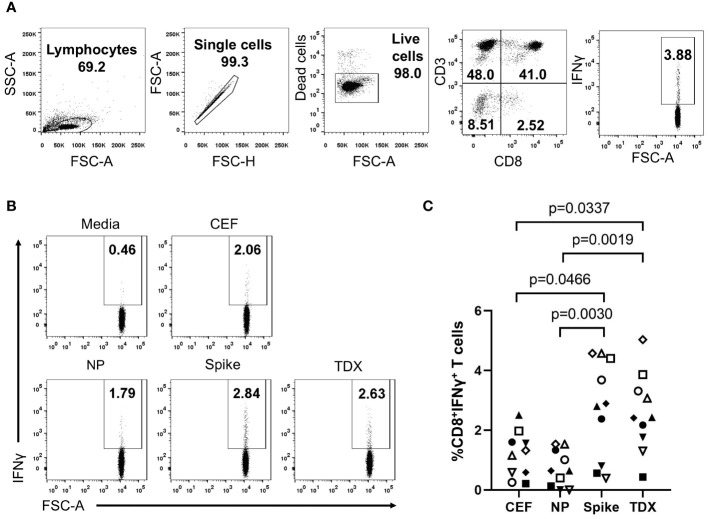
Existing T cell responses to SARS-CoV-2 peptide pools. PBMCs from 10 healthy subjects were stimulated with SARS-CoV-2 peptide pools (Spike, NP and TDX), CEF pool or media (complete RPMI with 0.3% DMSO) during 16 hours and CD8^+^ T cell responses detected by intracellular IFNγ staining assays **(A)** Gating strategy for the detection of intracellular IFNγ expression within the CD8^+^ T cell population by flow cytometry using a representative PBMC sample stimulated with the TDX peptide pool. IFNγ^+^CD3^+^CD8^+^ cells were identified after the following steps: a) Adequate adjustment of the gate of the lymphocytes using the light scatter parameters (FSC and SSC-A), b) Exclusion of doublets with the identification of singlets improving the accuracy of the analysis, c) Selection of the viable lymphocytes, d) Identification of the CD3^+^CD8^+^ cell subset and e) IFNγ^+^ cells within the CD3^+^CD8^+^ cells. IFNγ^+^ gate was set after FMO stainings with PHA-L stimulation (see **Supplementary Figure S1**). **(B)** Representative dot plot showing IFNγ^+^ cells on CD3^+^CD8^+^ gated cells in response to the different stimuli (Media, CEF, Spike, NP and TDX). Percentage of IFNγ^+^ cells are indicated **(C)** Graph depicting the percentage of CD8^+^ T cells expressing IFNγ (y-axis) in response to peptide pools (x-axis) after subtracting response to media (n = 10). Individual values are plotted. Statistically significant differences were obtained by applying Kruskal-Wallis tests. Significant differences are indicated and *p*-values shown.

Given the dimensions of COVID-19 pandemics and vaccination programs, SARS-CoV-2 specific memory T cells are now present in most individuals. Therefore, we first determined existing T cell recall responses to the TDX pool using PBMCs from 10 subjects (healthy blood donors) and compared them with those to SARS-CoV-2 peptide pools from spike (Spike pool) and nucleocapsid phosphoprotein (NP pool). To that end, we stimulated PBMCs with the noted peptide pools for 16 hours and subsequently analyzed intracellular IFNγ expression in CD8^+^ T cells by flow cytometry. As controls, we stimulated PBMCs with CEF pool and media alone (0.3% DMSO). In these experiments, we surely detect responses by memory CD8^+^ T cells although effector T cells could also respond in the case of recent vaccination or infection. We found dominant and statistically significant memory CD8^+^ T cells recall responses to TDX and Spike pools compared to CEF and NP pool (**Figure 1**). Moreover, all 10 subjects have detectable responses to the TDX pool, confirming the high population coverage of the CD8^+^ T cell epitopes included in the pool, outnumbering those responding to other peptide pools, including the Spike peptide pool. However, overall there was no statistical difference between the detected T cell recall responses to TDX and Spike pools. The detection of dominant and prevalent memory/effector responses to SARS-CoV-2 spike protein in the studied subjects is likely the result of COVID-19 vaccination. COVID-19 vaccines rely on inducing immunity to SARS-CoV-2 Spike protein (4) and over 85% of people in Spain aged 12 and above are fully vaccinated against COVID-19 (60). The TDX pool does also include 8 CD8^+^ T cell epitopes from SARS-CoV-2 spike protein but only 4 of them lie within the regions covered by the Spike peptide pool (underlined in **Table 1**). Therefore, it is unlikely that these epitopes can fully account for the comparable memory/effector T cell responses to TDX pool and Spike pool. Hence, the strong memory/effector T cell responses to TDX pool detected in most subjects are likely the result of SARS-CoV-2 infections and may also be impacted by pre-existing cross-reactive memory T cells elicited by vaccines with tetanus-diphtheria antigens. However, T cells are cross-reactive by nature and T cell immunity dynamic, which makes challenging to identify the source of pre-existing SARS-CoV-2 cross-reactive memory T cells. Therefore, in this work, we resorted to non-antigen experienced naive T cells, and examined whether Td-stimulations could activate them to respond to the SARS-CoV-2 TDX pool.

### 3.3 Td-stimulated responses of naive T cells to SARS-CoV-2 TDX pool

**Figure 2:**
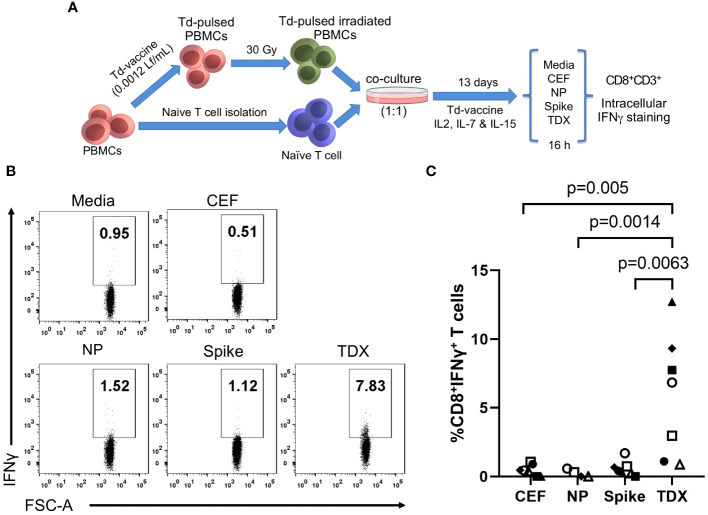
Cross-reactive responses of naive T cells stimulated with Td vaccine to SARS-CoV-2 peptide pools. **(A)** Experimental design to stimulate/prime T cells against Td vaccine. PBMCs were pulsed with Td vaccine (0.0012 Lf/ml of diphtheria toxoid) and homogenously irradiated at 30 Gy. Td-pulsed irradiated PBMCs were co-cultured with autologous naive T cells (ratio 1:1) for 13 days in the presence of IL-2, IL-7, IL-15 and Td vaccine (details in Methods). Subsequently, T cell responses to peptide pools (TDX pool, Spike pool, NP pool and CEF pool) and media (0.3% DMSO) were determined by intracellular IFNγ staining assays. IFNγ positive gate was set after FMO staining with PHA stimulation. **(B)** Representative dot plot showing the IFNγ^+^CD8^+^ T cells after stimulation with the relevant peptide pools **(C)** Percentage of CD8^+^ T cells expressing IFNγ after subtracting value from control media. Individual values were plotted (n = 7, except for NP with n = 5). Statistically significant differences were obtained by applying Kruskal-Wallis tests. Significant differences are indicated and *p*-values shown.

Tetanus-diphtheria vaccines must be capable of priming T cells cross-reactive with SARS-CoV-2 to have had an impact in the existing T cell immunity to the virus. To verify this point, we resorted to *in vitro* immunizations in which we stimulated antigen-inexperienced naive T cells from 7 subjects (healthy blood donors) with autologous irradiated PBMCs pulsed with Td vaccine, and then analyzed responses to SARS-CoV-2 peptide pools (**Figure 2A**). We found that Td vaccine can be toxic to cells and so we first worked out a dose of Td vaccine that was not toxic and foster proliferation of T cells in PBMCs (See Methods for details). As a result, we selected a dose of Td vaccine containing 0.0012 Lf/ml of diphtheria toxoid (**Supplementary Figure S2**) to pulse irradiated PBMCs. To enable priming conditions, autologous naive T cells were co-cultured with irradiated Td-pulsed PBMCs for 13 days in the presence of Td-vaccine, IL-2, IL-7 and IL-15 (details in Methods). Naive T cells used in these experiments were purified from PBMCs and had a purity of over 91% (**Supplementary Figure S3**). Subsequently, we investigated the responses of Td-stimulated T cells to SARS-CoV-2 peptide pools (Spike, NP and TDX), as previously described by intracellular IFNγ staining assays (details in Methods). As controls, the responses of Td-stimulated T cells to media and CEF pool was also determined. As shown in **Figures 2B, C**, Td-primed T cells from all subjects responded strongly to TDX pool (n = 7), while responses to Spike pool, CEF pool and NP pool were seldom detected (**Figure 2C**). It is worth noting that naive T cells stimulated with irradiated PBMCs in the absence of Td vaccine did not respond to TDX (see **Supplementary Figure S4**).

**Figure 3:**
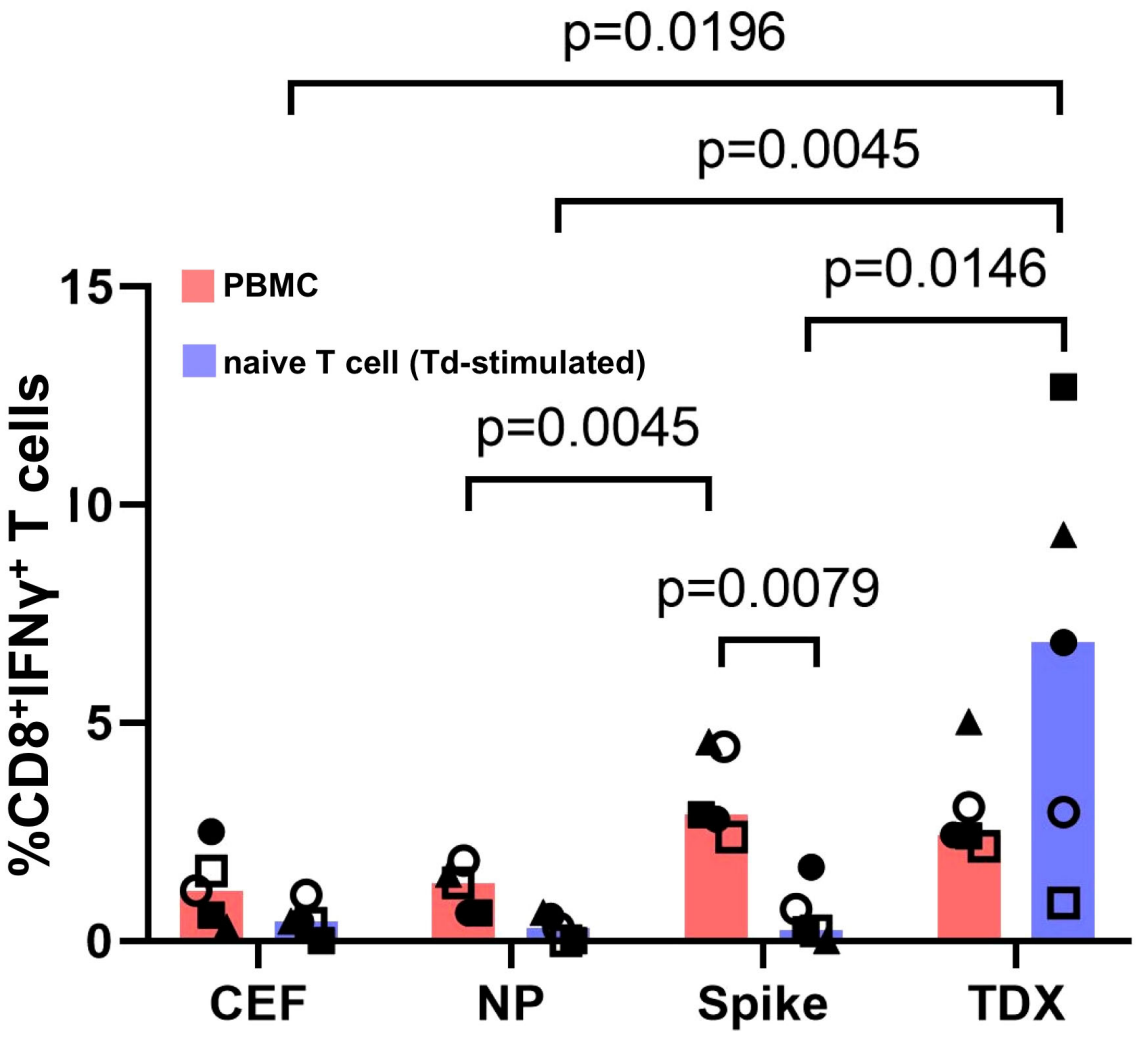
Responses to SARS-CoV-2 peptide pools from matched PBMCs and Td-stimulated native T cells. T cell responses to SARS-CoV-2 peptide pools (NP, Spike and TDX) and CEF pool determined using PBMCs (pink) and Td-stimulated naive T cells (blue) from the same five subjects. PBMCs and Td-stimulated naive T cells were incubated with peptide pools or media for 16 hours and responses were detected by intracellular IFNγ staining assays. All responses (value from media control subtracted) are plotted and bars represent median values. Statistically significant differences between conditions are indicated and *p*-values shown. Kruskal-Wallis tests were used for comparing responses to peptide pools from PBMC recall and Td-stimulated naive T cells. Wilcoxon signed-rank tests were carried out to compare T cell responses between PBMCs and Td-stimulated naive T cells in the same individuals to the same peptide pools.

The fact that Td-stimulated naive T cells responded only to SARS-CoV-2 CD8^+^ T cell epitopes that were anticipated as cross-reactive (TDX pool) with comparable strength than existing memory/effector T cells is truly outstanding. One could wonder if Td-stimulated T cell responses are due to contaminating TDX-specific memory/effector T cells or differentiated effector T cells (CD45RA^+^ TEMRA cells). However, this scenario is very unlikely. On the one hand, naive T cells used in the experiments were highly enriched (see **Supplementary Figure S3**) and did not respond to the TDX pool prior to Td stimulation (**Supplementary Figure S4**). On the other hand, if such contamination had occurred, Td-stimulated naive T cells should have also responded to the Spike pool but they did not. Further support of Td-priming of T cells cross-reactive with SARS-CoV-2 TDX pool is very noticeable in those individuals in which T cell responses were measured using PBMCs and Td-stimulated naive T cells (**Figure 3**). Statistical differences in matched responses to the different peptide pools mirrored those described previously, but were fewer given the smaller sample size (n = 5). Td-stimulated naive T cells only responded to the TDX pool. Moreover, it is worth noting that naive T cells from individuals with weak memory/effector T cell recall responses to the TDX pool responded strongly to this peptide pool after Td-stimulation. At the same time, Td-stimulated naive T cells from individuals with strong memory/effector T cell responses to the Spike pool did not respond to the Spike pool, only to the TDX pool. Overall, these results strongly support that Td vaccine can prime T cells to precisely recognize SARS-CoV-2-specific CD8^+^ T cell epitopes that were anticipated as cross-reactive with tetanus-diphtheria vaccines.

### 3.4 Immunization of mice with Td vaccine induces SARS-CoV-2 cross-reactive T cells

**Figure 4:**
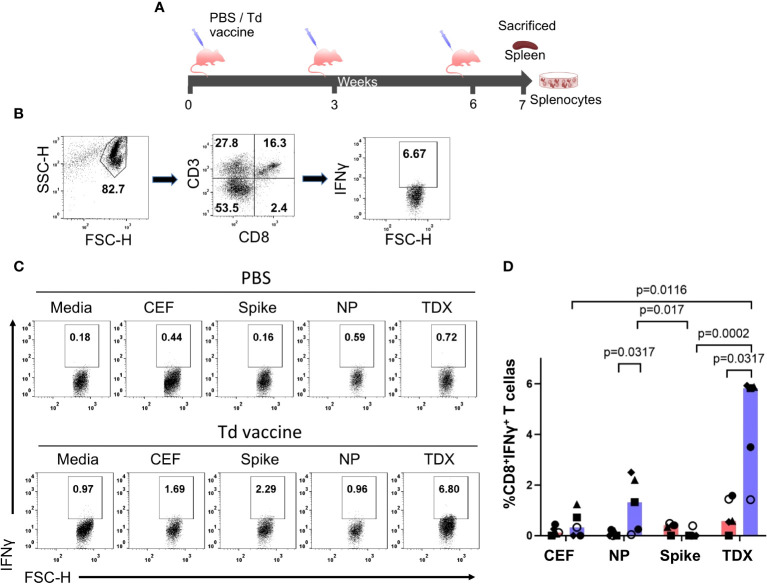
Cross-reactive T cell responses to SARS-CoV-2 peptide pools in Td-vaccinated mice. **(A)** Immunization schedule. Mice were immunized intramuscularly (IM) at 3-week intervals with Td vaccine (0.1 Lf diphtheria toxoid)(Td-vaccine group, n = 5) or PBS (PBS control group, n=5). Mice were sacrificed seven days after the last immunization, spleens were collected and splenocytes prepared and responses to SARS-CoV-2 peptide pools (NP, Spike and TDX), CEF pool and media (complete RPMI with 0.3% DMSO) after 36-hour stimulations determined by intracellular IFNγ staining assays **(B)** Gating strategy for intracellular IFNγ detection within the CD8^+^ T cell population by flow cytometry using mouse splenocytes stimulated with the TDX peptide pool. IFNγ^+^CD3^+^CD8^+^ cells were identified after the following steps: a) Adequate selection of cells using the light scatter parameters (FSC, SSC), b) Selection of CD3^+^CD8^+^ cells, c) Selection of IFNγ^+^ cells within the CD3^+^CD8^+^ cells. IFNγ^+^ gate set after FMO stainings with PHA-L stimulation (see **Supplementary Figure S1**) **(C)** Representative dot plot showing IFNγ^+^CD8^+^ T cells responding to SARS-CoV-2 peptide pools (TDX, Spike and NP), CEF pool or media in both, PBS and Td-vaccine groups. **(D)** Percentage of CD8^+^ T cells producing IFNγ (value from control media subtracted) in both, PBS control group (pink) and Td-vaccine group (blue). All values are plotted and bars represent median values. Statistically significant differences between responses are shown with *p-*values. Kruskal-Wallis tests were used for comparing T cell responses to peptide pools in Td-vaccinated and control mice. Mann-Whitney U tests were carried out to compare T cell responses to the same peptides (CEF, NP, Spike and TDX pools) between Td-vaccinated mice and control mice.

We also analyzed CD8^+^ T cell cross-reactivity to SARS-CoV-2 peptide pools in C57BL/6J mice immunized with Td vaccine. C57BL/6J mice express two H2 class I alloantigens, H2-Kb and H2-Db, that are predicted to present 5 of the cross-reactive CD8^+^ T cell epitopes (**Table 1**). We immunized mice with 3 IM injections of Td vaccine (0.01 Lfu of diphtheria toxoid)(n = 5) or PBS (control group, n = 5) at 3-week intervals, and sacrificed them one week after the last immunization to isolate splenocytes (**Figure 4A**). Appropriated immunization of mice with Td vaccine was confirmed by the detection of high levels of tetanus toxoid–specific IgG in blood serum (**Supplementary Figure S5**). Subsequently, we incubated splenocytes from Td-vaccinated and control mice for 36 hours with SARS-CoV-2 TDX, Spike and NP peptide pools. As controls, splenocytes were incubated with CEF pool and media (RMPI with 0.3% DMSO). Subsequently, we analyzed IFNγ production in CD8^+^ T cells by flow cytometry (**Figures 4B, C**). We observed that Td-vaccinated mice responded strongly to the TDX pool, with up to 8-fold increase in IFNγ-producing CD8^+^ T cells compared to non-vaccinated mice. Moreover, the response to the TDX pool in Td-vaccinated mice was significantly larger than that to CEF and Spike peptide pools, which were negligible. It is worth noting that Td-vaccinated mice also exhibited significant responses to the NP pool (**Figure 4C**). Overall, these results clearly show that Td-vaccination of mice induces SARS-CoV-2 specific CD8^+^ T cells recognizing the selected epitopes with similarity with tetanus-diphtheria vaccine antigens.

## 4 Discussion

There is increasing evidence that pre-existing cross-reactive T cell immunity contributes to protect against SARS-CoV-2 infection (33). Given the structural similarity between SARS-CoV-2 and ccHCoVs, it has become widely accepted that ccHCoVs are the sources of pre-existing cross-reactive immunity to SARS-CoV-2 (22). That ccHCoVs cause seasonal infections in humans with higher incidence in children (35) supports this view. However, evidence of exposure to ccHCoVs is seen in 95% of adults (35) and why cross-reactive immunity from ccHCoVs could be more protective in children than in young adults is open to speculation. On the other hand, it has also been reported that prior infection by seasonal ccHCoVs does not prevent SARS‐CoV-2 infection in children (61). Moreover, it has been noted that pre-existing SARS-CoV-2 cross-reactive T immunity cannot be solely explained by ccHCoVs infections (62). Hence, there must be additional sources of T cell cross-reactivity to SARS-CoV-2.

T cell cross-reactivity is actually quite common. During maturation in the thymus, individual T cells are required to recognize numerous peptides presented by the same MHC molecules, which render them cross-reactive by nature (29). In fact, it has been reported that a single T cell receptor can recognize about a million different peptides (63). Therefore, it is not surprising that T cell cross-reactivity to SARS-CoV-2 had been found beyond ccHCoVs, reaching to unrelated viruses (43, 44), vaccines such as MMR and Tdap (50), and bacteria (42). However, until now and to our knowledge none of these candidates have been shown to prime antigen-inexperienced naive T cells cross-reacting with SARS-CoV-2. Such priming ought to be necessary to regard a candidate as responsible of pre-existing cross-reactive T cell immunity. Following the tracks of an early study proposing tetanus-diphtheria vaccine antigens as sources of protective cross-reactive immunity to SARS-CoV-2 (47, 48), in this exploratory work we show proof that Td vaccine can prime T cells cross-reacting with known SARS-CoV-2-specific CD8^+^ T cell epitopes.

Vaccines with tetanus-diphtheria toxoids include hundreds of additional antigens (51–53) and are widely used. During infancy children receive three immunizations with DTP vaccine (diphtheria and tetanus toxoids combined with antigens from *Bordetella persussis*), alone or combined with other vaccines like Polio vaccine, hepatitis B vaccine and conjugated *Haemophilus influenzae* type B vaccine (Hib vaccine) (64). Children receive one additional DTP vaccination at 4–6 years of age and a boost in puberty with versions containing a lower dose of diphtheria antigens (d), with or without a low dose of antigens from acellular *B. pertussis* (ap): Tdap or Td vaccine, respectively (64). Td vaccines are also given in the case of severe or unclean wounds (64) and immunization with Tdap vaccine is recommended for pregnant women (65). Moreover, conjugated pneumococcal vaccines and Hib vaccines also use diphtheria or tetanus toxoids for conjugation (66, 67). In sum, T cell immunity and memory to tetanus-diphtheria vaccine antigens develop early during childhood through repeated vaccinations and can be present in adults (64, 68).

Memory T cells elicited by tetanus-diphtheria antigens ought to imprint existing T cell responses to SARS-CoV-2, provided that cross-recognition occurs. Therefore, we sought for evidence of cross-reactivity from tetanus-diphtheria vaccine antigens in experimentally verified SARS-CoV-2 CD8^+^ T cell epitopes, recognized by humans during the course of infection. We identified 66 SARS-CoV-2-specific CD8^+^ T cell epitopes sharing sequence similarity (≤ 20% Levenshtein distance edits) with tetanus-diphtheria vaccine antigens, suggesting cross-reactivity. Subsequently, we investigated T cell responses to 25 of these potentially cross-reactive SARS-CoV-2 CD8^+^ T cell epitopes (TDX pool) that were selected for covering most of SARS-CoV-2 antigens and for their presentation by 13 distinct HLA I molecules (**Table 1**). In the studied subjects (healthy blood donors), we observed dominant memory/effector T cell responses to the selected, potentially cross-reactive, SARS-CoV-2 CD8^+^ T cell epitopes (TDX pool) that were comparable to those against a peptide pool covering the Spike protein (**Figures 1**, **3**). We had no information on COVID-19 vaccination status of participants or if they have been infected by SARS-CoV-2 but presumably all participants had been vaccinated for COVID-19 and passed the infection. In this context, strong and prevalent memory T cell recall responses to SARS-CoV-2 spike peptides are likely due to T cell immunity elicited by both, COVID-19 vaccinations and infection. On the other hand, the comparable memory/effector T cell responses to the TDX pool can be attributed to SARS-CoV-2 infections and may also be compatible with pre-existing T cell immunity elicited by tetanus-diphtheria vaccine antigens. This latter possibility is supported by the fact that stimulating naive T cells with autologous irradiated PBMCs pulsed with Td vaccine resulted in T cells that responded strongly to TDX pool but not to control peptides, including other SARS-CoV-2 peptide pools (**Figures 2**, **3**). We are aware that we worked with a very small donor sample size, which could limit the generalizability of our findings. However, our results are not only statistically significant, but also quite compelling. Td-primed T cells from every single donor responded to TDX pool using Td-stimulated naive T cells, while the same cells did not respond to other peptide pools. It has been reported that immunogenic CD8^+^ T cell epitopes are characterized by the presence of short motifs that are highly represented in the human proteome (69). Interestingly, the 25 SARS-CoV-2 CD8^+^ T cell epitopes included in the TDX pool displayed an average identity to human proteins of 66.1 ± 5.9, which may indicate the presence of such motifs. None of the selected epitopes were however identical to human proteins (see **Supplementary Dataset 2**).

Similar results were reproduced *in vivo* using C57BL/6J mice immunized with Td vaccine. Td-vaccination elicited in mice induced SARS-CoV-2 cross-reactive CD8^+^ T cells responding strongly to TDX pool (**Figure 4**). However, unlike Td-primed T cells from humans, Td-vaccinated mice also responded to SARS-CoV-2 NP pool. All together these results show that i) vaccines with tetanus-diphtheria toxoids can be a priming source of cross-reactive T cell immunity to SARS-CoV-2 and ii) SARS-CoV-2-specific CD8^+^ T cell epitopes in TDX pool are indeed cross-reactive with tetanus-diphtheria antigens, as predicted by Levenshtein edit distances. We should point that not necessarily all SARS-CoV-2-specific CD8^+^ T cell epitopes in TDX pool are cross-reactive with tetanus-diphtheria antigens. Conversely, there might be may other SARS-CoV-2 T cell epitopes cross-reactive with tetanus-diphtheria vaccine antigens. Indeed, the TDX pool only included 25 of the 66 SARS-CoV-2 CD8^+^ T epitopes that were predicted to be cross-reactive with tetanus-diphtheria antigens (**Supplementary Dataset 2**). Moreover, T cell cross-reactivity is not always predictable and epitopes without or very little sequence similarity can be cross-reactive (70). Therefore, further work is required to confirm individually SARS-CoV-2 cross-reactive T cell epitopes, as well as their counterparts in Td vaccines. Selecting SARS-CoV-2 CD8^+^ T cell epitopes related by similarity with tetanus-diphtheria vaccine antigens is not an unbiased method to detect cross-reactivity, but it provides an objective and reproducible manner to detect cross-reactive epitopes. Therefore, we believe that this same approach could be applied to investigate cross-reactivity between other vaccines and pathogens, saving much time and resources. Moreover, our approach to detect cross-reactive epitopes could be enhanced by taking in consideration if amino acid edits occur in anchor or exposed amino acid positions. However, it is worth noting that cross-reactivity may involve changes in both, anchor or non-anchor positions (71). Taking in consideration HLA binding profiles of epitopes and matching peptides could also serve to improve the selection of potentially cross-reactive T cell epitopes.

Through a completely different approach, Mysore et al. (50) identified T cell cross-reactivity between SARS-CoV-2 and Tdap vaccine, which contains tetanus and diphtheria antigens. In their study, Mysore et al. stimulated total human T cells with MMR or Tdap vaccines on the one hand and on the other with SARS-CoV-2 antigens, and subsequently carried out single cell RNA sequencing and TCR clonotyping. The authors found that T cells stimulated with these vaccines and SARS-CoV-2 antigens displayed overlapping TCRs (CD3 regions), which is sign of cross-reactivity. In addition, these authors found that COVID-19 disease severity was reduced in Tdap-vaccinated individuals by 20%–23%. However, Mysore et al. did not show that Tdap vaccine can prime/stimulate naive T cells nor investigated the responses of Tdap activated T cells to SARS-CoV-2 epitopes. Although we determined SARS-CoV-2 T cell epitopes that are cross-reactive with Td vaccines, we did not explore if these particular epitopes mediate protective immunity. Moreover, we did not characterize cross-reactive T cells but our results will facilitate the design of tetramers to label antigen-specific CD8^+^ T cells and show the presence of cross-reactive T cells.

## 5 Conclusions

Naive T cell cells stimulated *in vitro* with Td vaccine are cross-reactive with known SARS-CoV-2 CD8^+^ T cell epitopes sharing similarity to tetanus-diphtheria vaccine antigens. Similarly, C57BL/6J mice immunized with Td vaccine respond to SARS-CoV-2-specific CD8^+^ T cell epitopes. Therefore, we conclude that tetanus-diphtheria vaccines can prime SARS-CoV-2 cross-reactive T cells, likely shaping existing T cell responses to the virus. Whether the selected SARS-CoV-2 CD8^+^ T cell epitopes that are cross-reactive tetanus-diphtheria mediate protective immunity remains to be determined. We only studied immune responses in a controlled environment and the clinical relevance of these findings needs further investigation. Nonetheless, pre-existing SARS-CoV-2 cross-reactive memory T cells have been shown to be protective (33) and there is already mounting evidence indicating that vaccines with tetanus-diphtheria antigens can make a contribution: 1) as shown here, naive T cells stimulated with tetanus-diphtheria vaccine respond strongly to SARS-CoV-2 epitopes; 2) tetanus-diphtheria vaccinations are associated with lower chances of developing severe COVID-19, even in the elderly (49); and 3) T cell immunity to tetanus-diphtheria antigens wanes with age (72), correlating inversely with the incidence of SARS-CoV-2 infections (46). Currently, immunizations with Td vaccine are recommended for adults every 10 years and, although we cannot correlate the data presented here with real clinical implications, our results strongly support staying up to date with Td boosters.

## Data availability statement

The original contributions presented in the study are included in the article/**Supplementary Material**. Further inquiries can be directed to the corresponding author.

## Ethics statement

The studies involving humans were approved by Comité de Evaluación de Investigación y Docencia del Centro de Transfusión de Madrid. The human samples used in this study were acquired from Buffy coats from healthy blood donors. Written informed consent for participation was not required from the participants or the participants’ legal guardians/next of kin in accordance with the national legislation and institutional requirements. The studies were conducted in accordance with the local legislation and institutional requirements. The participants provided their written informed consent to participate in this study. The animal study was approved by Ethics Board Committee at the Universidad Complutense de Madrid. The study was conducted in accordance with the local legislation and institutional requirements.

## Author contributions

SAF: Formal analysis, Methodology, Validation, Visualization, Writing – review & editing. HFP-P: Formal analysis, Methodology, Validation, Visualization, Writing – original draft. TF: Formal analysis, Methodology, Validation, Visualization, Writing – review & editing. MG-P: Methodology, Writing – review & editing. JR: Formal analysis, Methodology, Writing – review & editing. PAR: Conceptualization, Funding acquisition, Investigation, Methodology, Software, Supervision, Writing – original draft, Writing – review & editing.

## Supplementary material

Supplementary Data Sheet 1 Excel file including 1153 experimentally verified SARS-CoV-2-specific CD8^+^ T cell epitopes reported to be targeted in humans infected by the virus.

Supplementary Data Sheet 2 Excel file including 66 experimentally verified SARS-CoV-2-specific CD8^+^ T cell epitopes related by similarity with tetanus-diphtheria vaccine antigens, as judged by Levenshtein edit distances.

Supplementary Figure S1 Fluorescence minus one (FMO) controls for intracellular cytokine staining assays.

Supplementary Figure S2 T cell proliferation upon Td vaccine stimulation.

Supplementary Figure S3 Quality control of isolated naive T cells.

Supplementary Figure S4 Control T cell responses.

Supplementary Figure S5 Tetanus Toxoid (TT)– specific IgG.
