# Supplementary material for: Tetanus-diphtheria vaccine can prime SARS-CoV-2 cross-reactive T cells
Source: Front Immunol. 2024 Jul 18;15:1425374. doi: 10.3389/fimmu.2024.1425374 (PMC11291333; doi:10.3389/fimmu.2024.1425374)
Supplement: Supplementary Figure S1 — Fluorescence minus one (FMO) controls for intracellular cytokine staining assays. [file Image_1.pdf]

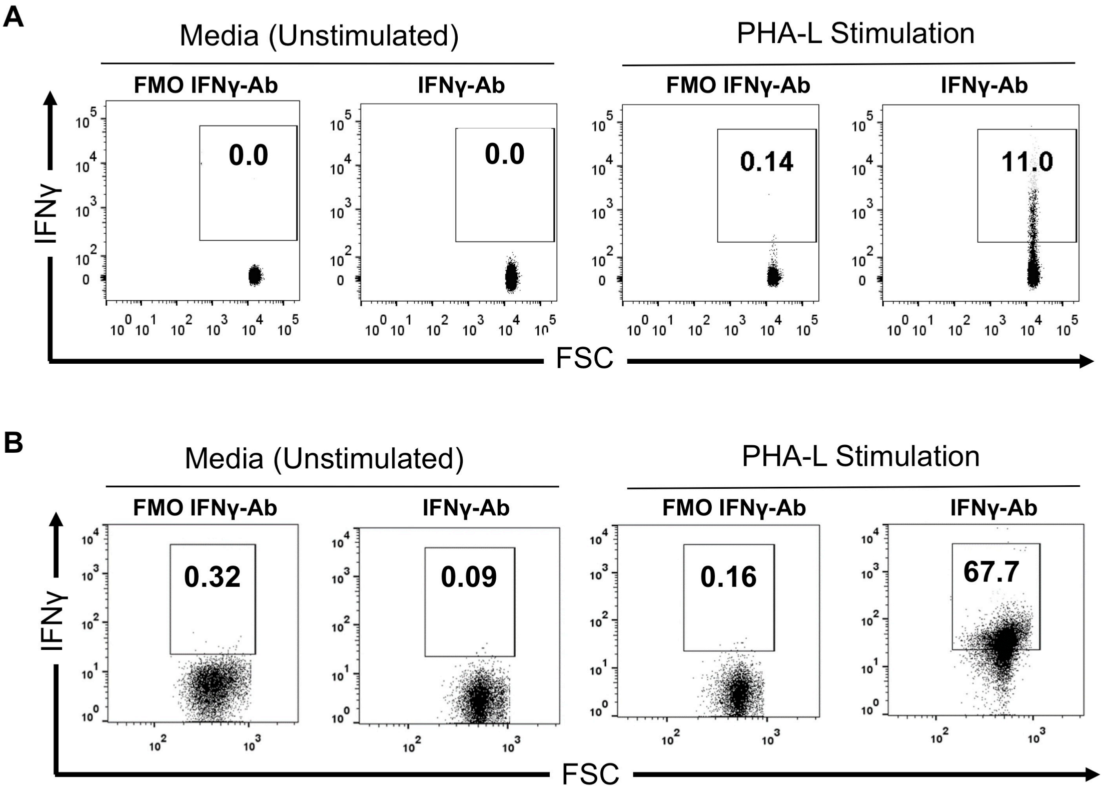

Supplementary Figure S1. Fluorescence minus one (FMO) controls for intracellular cytokine staining assays. IFNγ antibody FMO controls were determined within $CD8^+$ T cells in human and mice. (**A**) IFNγ FMO control for human. Human PBMCs were incubated for 16 hours (37ºC and 5% CO2) with media (Unstimulated) or PHA-L (5 µg/ml)(PHA-L Stimulation), stained extracellularly with anti-CD3 and anti-CD8 antibodies and intracellularly with anti-IFNγ antibody (IFNγ-Ab) or without (FMO IFNγ-Ab). Dot plot shows $IFN\gamma^+$ cells (gated on $CD3^+CD8^+$ cells) in the different conditions with the positive gate frame selected after the stimulation with PHA-L. Human samples were acquired in a BD Celesta cytometer (BD Biosciences). (**B**) IFNγ FMO control for mice. Mice splenocytes were incubated for 36 hours (37ºC and 5% CO2) with media (Unstimulated) or PHA-L (5 µg/ml)(PHA-L Stimulation), stained extracellularly with anti-CD3 and anti-CD8 antibodies and intracellularly with anti-IFNγ antibody (IFNγ-Ab) or without (FMO IFNγ-Ab). Dot plot shows $IFN\gamma^+$ cells (gated on $CD3^+CD8^+$ cells) in the different conditions with the positive gate frame selected after stimulation with PHA-L. Mice samples were acquired in a BD FacsCalibur cytometer (BD Biosciences). Numbers in each plot represents the percentage of positive IFNγ cells within $CD8^+$ T cells for each condition. All data was analyzed using FlowJo V10 Software (Treestar).
