# Supplementary material for: Tetanus-diphtheria vaccine can prime SARS-CoV-2 cross-reactive T cells
Source: Front Immunol. 2024 Jul 18;15:1425374. doi: 10.3389/fimmu.2024.1425374 (PMC11291333; doi:10.3389/fimmu.2024.1425374)
Supplement: Supplementary Figure S2 — T cell proliferation upon Td vaccine stimulation. [file Image_2.pdf]

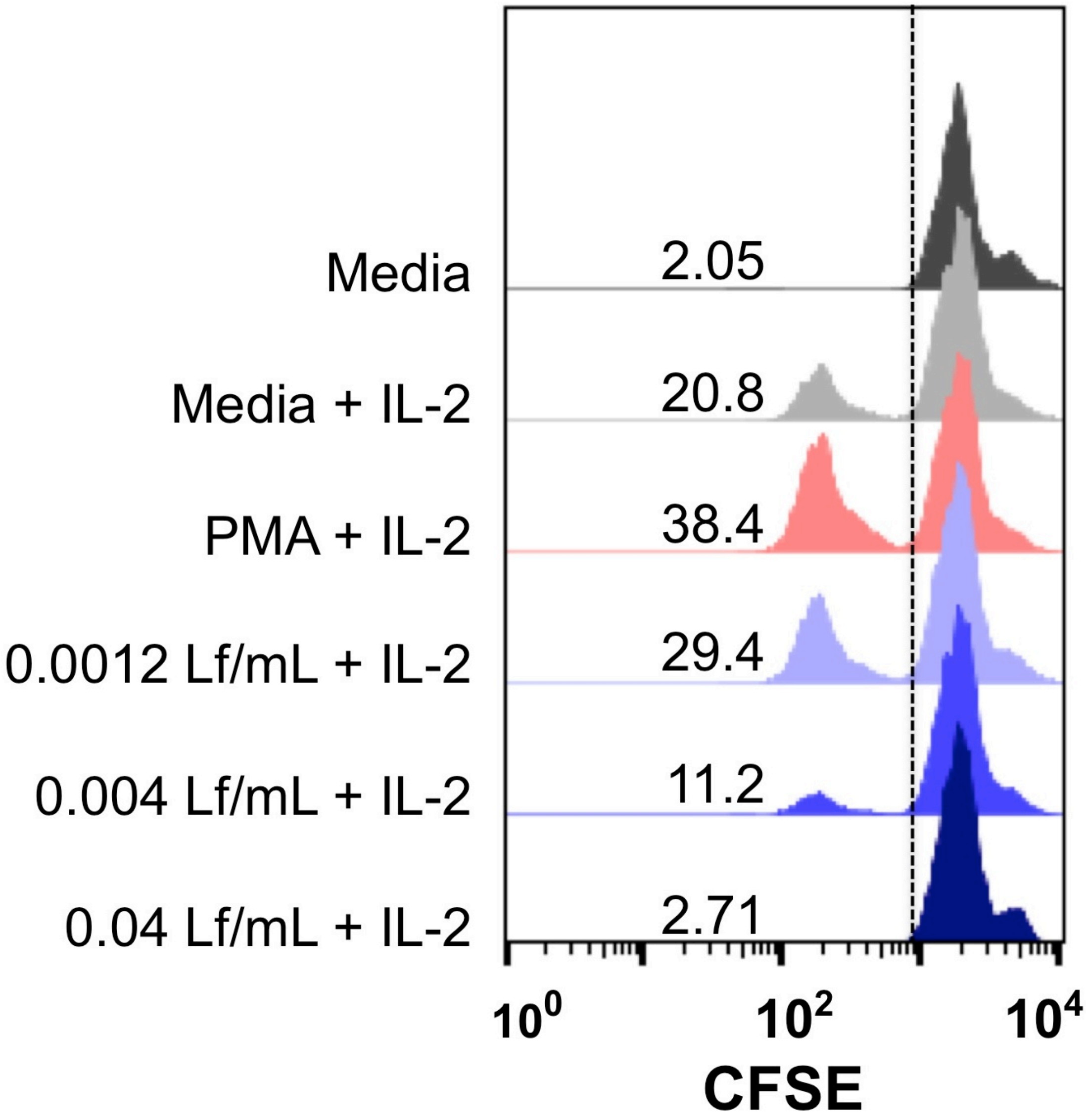

Supplementary Figure S2. T cell proliferation upon Td vaccine stimulation. CFSE-labeled PBMCs were stimulated with Td vaccine (0.0012, 0.004 and 0.04 Lf/ml of diphtheria toxoid), PMA (25 ng/mL) or media alone. All conditions were culture with 20 ng/mL of IL-2 for five days. Cells were stained with anti-CD3 antibody and cell proliferation on gated $CD3^+$ cells was analyzed by flow cytometry. The percentage of cells undergoing division is indicated in the histograms.
