# Supplementary material for: Tetanus-diphtheria vaccine can prime SARS-CoV-2 cross-reactive T cells
Source: Front Immunol. 2024 Jul 18;15:1425374. doi: 10.3389/fimmu.2024.1425374 (PMC11291333; doi:10.3389/fimmu.2024.1425374)
Supplement: Supplementary Figure S3 — Quality control of isolated naive T cells. [file Image_3.pdf]

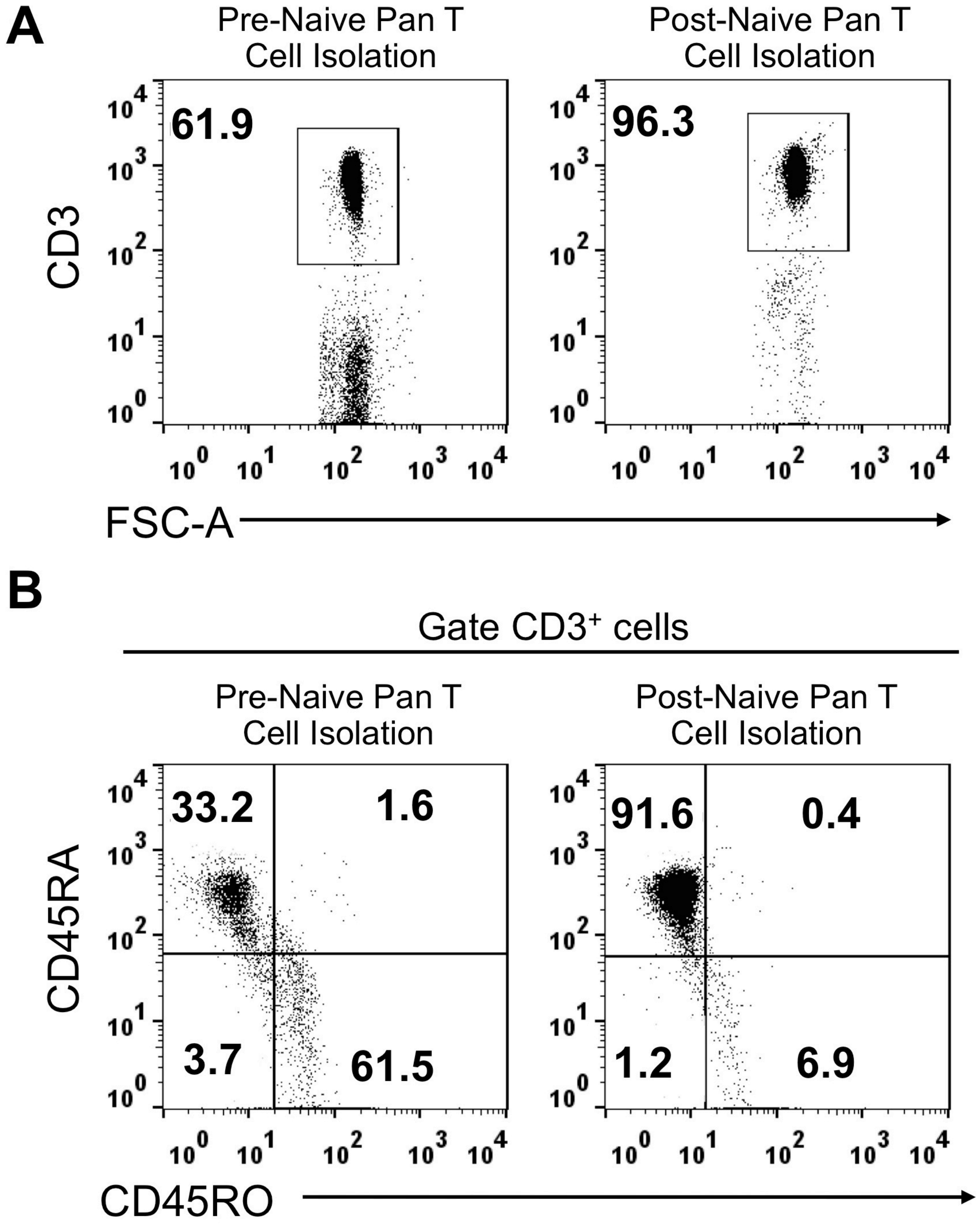

Supplementary Figure S3. Quality control of isolated naive T cells. Naive T cells were isolated from PBMCs using a magnetic separation kit. Cells before and after naive T cell isolation were stained with antibodies anti-CD3, anti-CD45RA and anti-CD45RO, and analyzed by flow cytometry. Expression of these markers was analyzed by flow cytometry. (**A**) Dot plot showing the percentage of $CD3^+$ cells before and after naive isolation from PBMCs. (**B**) Dot plot showing the percentage of $CD3^+CD45RA^+$ vs $CD3^+CD45RO^+$ cells before and after cell isolation.
