# Supplementary material for: Tetanus-diphtheria vaccine can prime SARS-CoV-2 cross-reactive T cells
Source: Front Immunol. 2024 Jul 18;15:1425374. doi: 10.3389/fimmu.2024.1425374 (PMC11291333; doi:10.3389/fimmu.2024.1425374)
Supplement: Supplementary Figure S4 — Control T cell responses. [file Image_4.pdf]

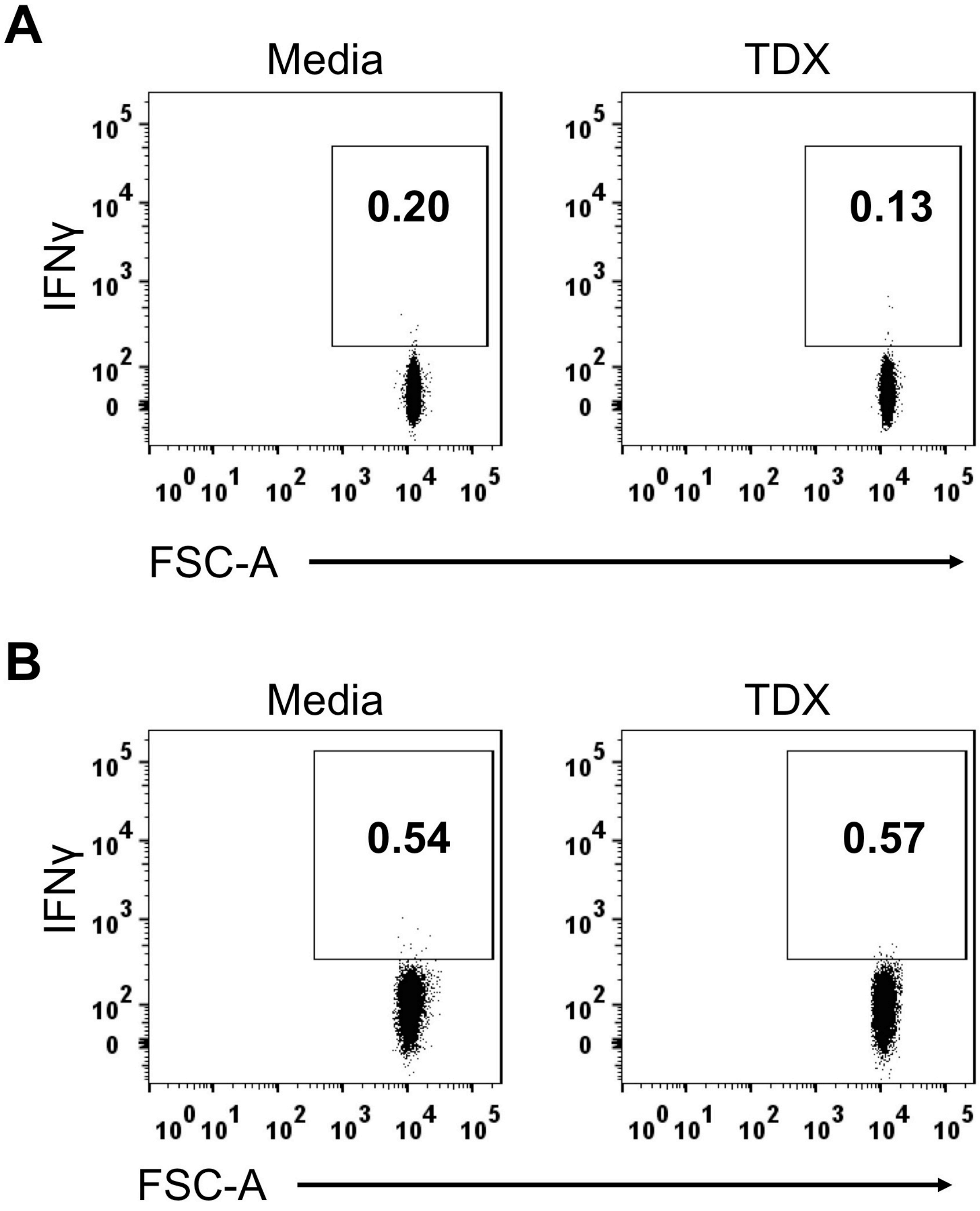

Supplementary Figure S4. Control T cell responses. (**A**) Dot plots showing IFNγ$^+$CD8$^+$ T cells after stimulating freshly isolated naive T cells with TDX pool for 16 hours. (**B**) IFNγ$^+$CD8$^+$ T cells resulting after culturing naive T cells with irradiated PBMCs without being pulsed/cultured with Td vaccine (non-pulsed). PBMCs were homogenously irradiated at 30 Gy. Non-pulsed irradiated PBMCs were co-cultured with autologous naive T cells (ratio 1:1) for 13 days without Td-vaccine and in the presence of IL-2, IL-7 and IL-15 (cytokines renewed every two days). After culture, cells were washed, rested, stimulated for 16 hours with TDX pool and subject to intracellular IFNγ staining assays.
