# Supplementary material for: Tetanus-diphtheria vaccine can prime SARS-CoV-2 cross-reactive T cells
Source: Front Immunol. 2024 Jul 18;15:1425374. doi: 10.3389/fimmu.2024.1425374 (PMC11291333; doi:10.3389/fimmu.2024.1425374)
Supplement: Supplementary Figure S5 — Tetanus Toxoid (TT)– specific IgG. [file Image_5.pdf]

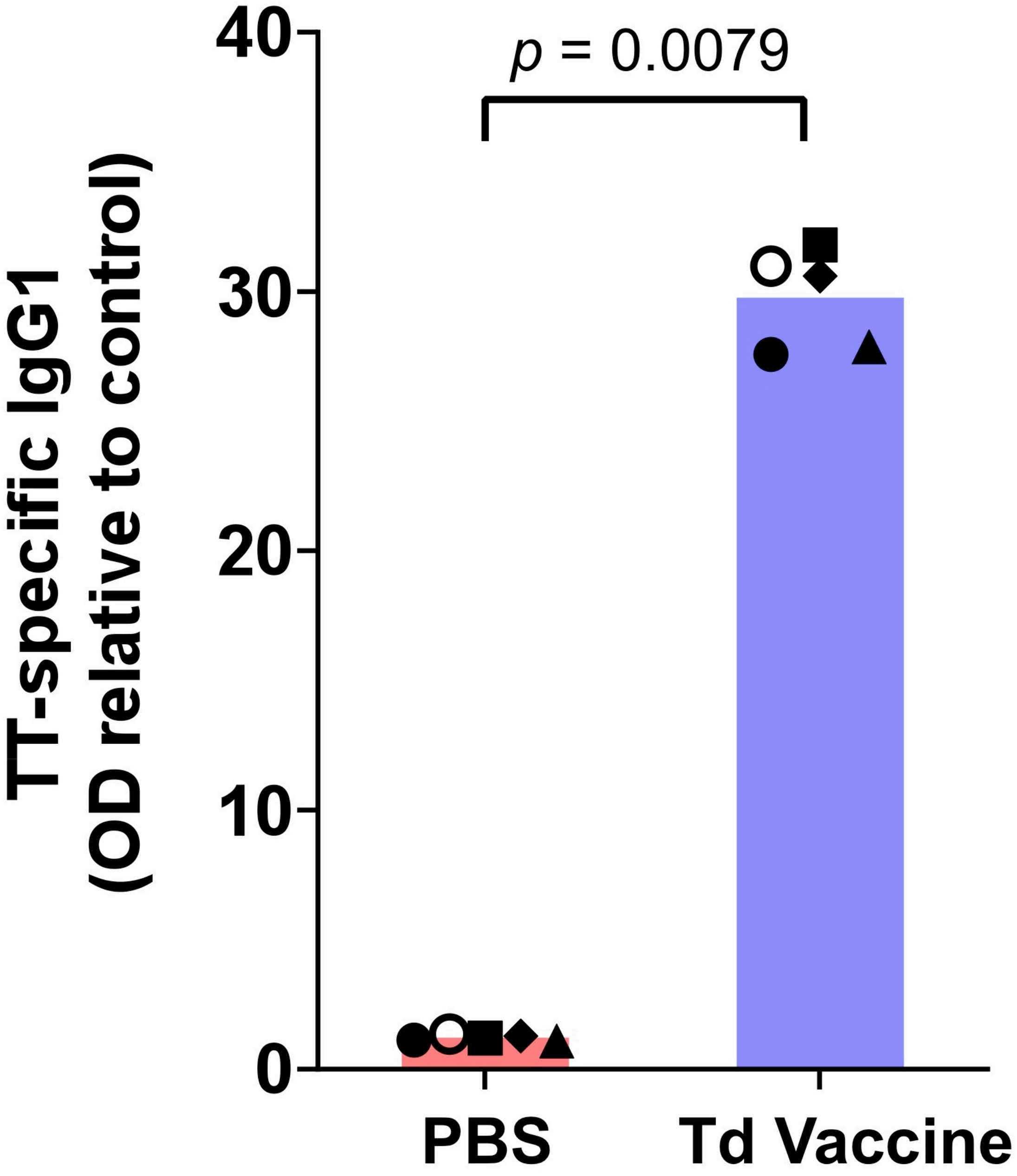

Supplementary Figure S5. Tetanus Toxoid (TT)–specific IgG. TT-specific IgG1 was measured in serum from Td-vaccinated (n = 5) and control mice (n = 5) by an indirect ELISA, 7 weeks after the initial vaccination. Control mice received PBS instead of Td vaccine. TT-specific IgG1 levels are plotted as optical density (OD) values determined in PBS and Td vaccine groups relative to blank controls. All values are plotted and bars represent median values. Statistically significant differences between the two groups are indicated, and *p*-values are shown. The Mann-Whitney U test was used to infer significance.
